# Cirques of the Southeastern Tibetan Plateau and Their Links to Climatic and Non-Climatic Factors

**DOI:** 10.3390/ijerph192013104

**Published:** 2022-10-12

**Authors:** Shengxian Li, Qian Zhang, Jiahan Wang

**Affiliations:** College of Geography and Environment, Shandong Normal University, Jinan 250358, China

**Keywords:** glaciation, glacier, paleoclimate, Quaternary, monsoon

## Abstract

Cirque morphology is used to reflect the patterns of paleoclimate, paleoglaciation, and landscape evolution. Cirque study has been conducted in the Gangdise Mountains of the southern Tibetan Plateau (TP) and the central TP (dominated by a weak Indian summer monsoon (ISM) or a continental climate). This study focused on the cirques in the southeastern TP, which is dominated by a strong ISM, to analyse the controlling factors on cirque morphology. A total of 361 cirques were mapped in the Taniantaweng Range of the southeastern TP, and their metrics were calculated. The results showed that the cirque sizes increased with temperature and decreased with precipitation, which may be due to the development of valley-type glaciers and the effect of non-climatic factors. The cirques tended to face NE, implying that they prefer leeward slopes, and they were under the ‘morning–afternoon’ effect. With altitude, the tendency of the cirque aspect shifted from N to SE, and the cirque size decreased. The former may indicate the ability of the high altitude to support cirque development on climatically unfavourable slopes; the latter may be due to the development of valley-type glaciers or insufficient space for cirque development. The cirque size and shape did not show statistical differences between aspects. The cirques on soft bedrocks had larger heights than those on hard bedrocks, indicating that soft bedrocks promote subglacial erosion. A comparison with the results of the western, central, and eastern sectors of the Gangdise Mountains and the central TP reveals that the strength of the ISM did not necessarily increase the cirque density but limited the cirque size on a regional scale. The CFA did not show a reverse relationship with precipitation, but it showed a positive correlation with the cirque Z_mean_, which implies that the CFA was greatly affected by altitude, and its distribution does not always reflect paleoclimatic patterns.

## 1. Introduction

**Figure 1:**
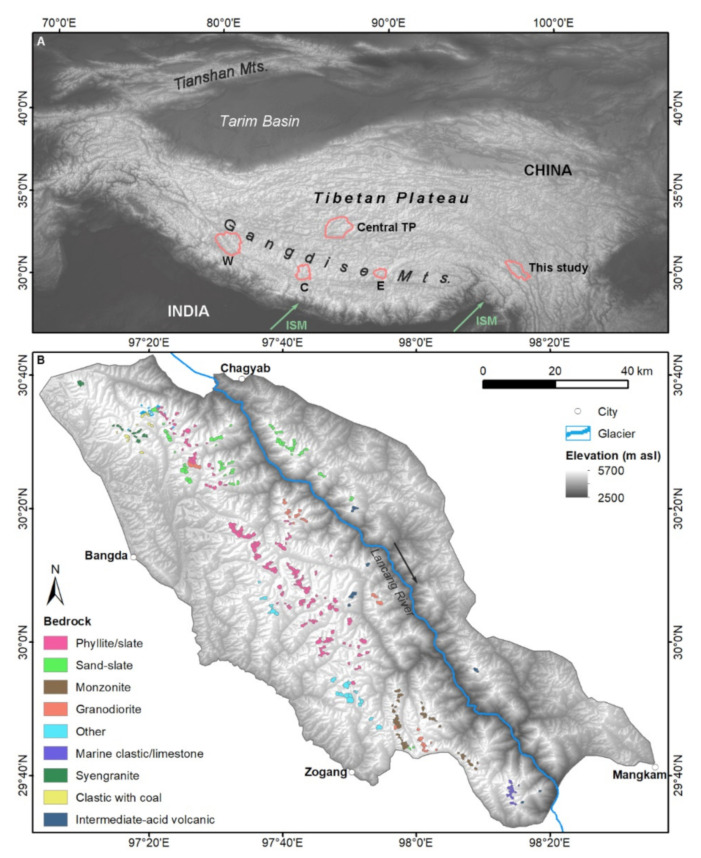
(**A**) Cirque study sites in the western, central, and eastern sectors of the Gangdise Mountains [7], the central TP [6], and this study; (**B**) cirque locations and bedrock types of the study area.

Cirques are present almost ubiquitously in formerly glaciated regions. They have been studied throughout the world [1,2,3,4,5,6,7], and these studies have shown that cirque morphology is linked to the paleoclimate. For example, a large cirque size indicates that former glaciers were dynamic or long-lasting; the cirque floor altitude (CFA) is an indicator of the former glacial equilibrium line altitude (ELA) and reflects information about the former moisture and wind directions; and the cirque aspect indicates information about the solar radiation, wind direction, and glacial extensions [2]. Many places on the Tibetan Plateau (TP) are difficult to reach due to their remoteness and extreme environment. In this case, cirques are good indicators for paleoclimate and paleoglaciation. The climatic domains of the TP can be categorised into a zone dominated by the Indian summer monsoon (ISM) in the south, a westerlies-dominated zone in the north, and a transitional zone lying in between (Figure 1) [8]. As the TP is characterised by various topographies, it has experienced various glaciations that differ from region to region. The cirques of the Gangdise Mountains and the central TP have been studied, and the results showed that increasing moisture limited the cirque density [6,7]. The effect of moisture on cirque size seemed to be dependent on the level of moisture, and moisture promoted cirque enlargement under low moisture and limited cirque enlargement when the moisture level surpassed a certain range [6]. In addition to climate, these studies found that topography and geology (e.g., altitude, orientation, and lithology) also affected cirque morphology, and sometimes these non-climatic factors played a dominant role [9,10,11]. Even though CFA is believed to indicate glacial ELA during glaciations [12,13,14,15], a recent study found that CFA was greatly controlled by non-climatic factors, and the efficacy of CFA as an ELA indicator was limited [16]. These studies added uncertainty to the efficacy of cirque morphology as a climatic indicator. To further analyse the relations of cirques and climatic/non-climatic factors, this study mapped the cirques in the northern part of Taniantaweng Range, southeastern TP, where the ISM dominates. This study is compared with the results of the Gangdise Mountains (their central-eastern part is dominated by a weak ISM, and the western part is dominated by a continental climate) and the central TP (dominated by a continental climate) to analyse the role of the ISM on cirque development. This study deepens the knowledge of a cirque’s reaction to monsoon at a regional scale and tests the efficacy of the cirque as a paleoclimatic indicator.

## 2. Study Area

**Figure 2:**
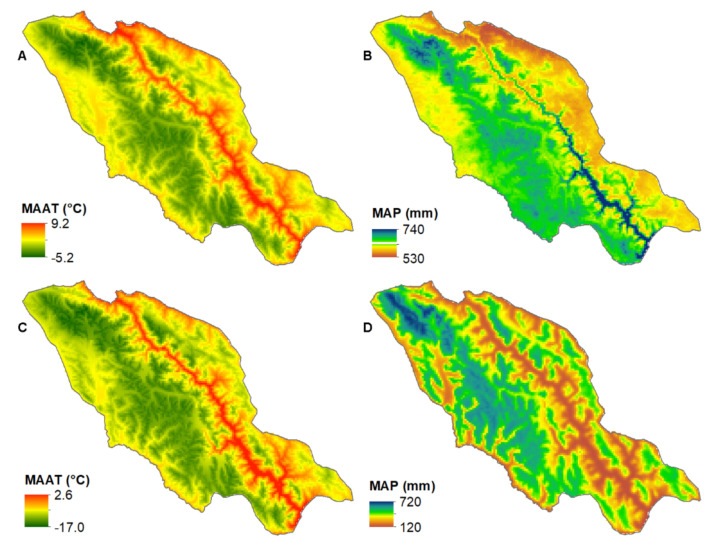
(**A**) Modern mean annual air temperature, (**B**) modern mean annual precipitation, (**C**) mean annual temperature and (**D**) mean annual precipitation during the Last Glacial Maximum. Data from WorldClim 2.1 (https://worldclim.org/; accessed on 20 July 2022) and PaleoClim (http://www.paleoclim.org/; accessed on 20 July 2022) [17,21].

The study area (29.5–30.7 °N, 97.0–98.6 °E) was located in the southeastern part of the TP, covering an area of ~8400 km^2^ (Figure 1). The Taniantaweng Range lay in the middle part of the study area, while the western and eastern parts were characterised by low relief. The study area is dominated by the ISM, with a mean annual air temperature ranging from –5.2 °C to 9.2 °C and mean annual precipitation ranging from 530 mm to 740 mm. From west to east, the mean annual air temperature increases, while the mean annual precipitation decreases (Figure 2A,B) (https://worldclim.org/; accessed on 20 July 2022) [17]. The northern part of the mapping domain was occupied by 20 contemporary glaciers, with areas ranging from 0.02 to 0.69 km^2^ [18] (Figure 1). Chronological studies have shown that this region experienced frequent glaciations during the marine isotope stages (MIS) 6, 3, 2, and 1 [19,20].

## 3. Methods

The cirques were mapped manually using the Google Earth image. Their length (L; m), width (W; m), height (H; m), area (km^2^), plan closure (°), profile closure (°), mean altitude (i.e., mean elevation of the topography within the cirque boundaries; Z_mean_; m asl), cirque floor altitude (CFA; m asl), mean slope gradient (S_mean_; °), and aspect were calculated (see Supplementary Material). The plan closure is the aspect range along the mid-elevation contour of a cirque [22], while the profile closure is the difference between the maximal headwall gradient and the minimal floor gradient [23,24]. Higher plan or profile closure values indicate the presence of better-developed cirques [23,24,25]. The CFA is defined as the minimum altitude within a cirque [26]. The vector mean (VM) and vector strength (VS) were calculated to indicate the cirque aspect tendency. The VM was defined as the direction of vector consultant by plotting the rock glacier aspect in a cumulated form [27], while the VS was the proportion between the length of the VM and the total length of individual vectors [28]. Two cirques were occupied by glaciers, which caused uncertainty for the calculation of their H, plan closure, profile closure, and CFA. To reduce such uncertainty, these metrics were calculated based on the subglacial topography, which was obtained by extracting the glacier thickness (derived from the dataset of Farinotti et al. [29]) from the modern surface. All metrics were calculated in ArcGIS 10.7 using the Automated Cirque Metric Extraction (ACME) toolbox [30], based on the Shuttle Radar Topography Mission (SRTM) 1 arc second digital elevation model (DEM) (https://earthexplorer.usgs.gov; accessed on 20 July 2022) under the framework of the Universal Transverse Mercator (UTM) 47 N projection. The cirque bedrock types were derived from a 1:1,500,000 geological map [31]. The impact of the altitude, slope, and bedrock type on the cirque morphology was analysed using Welch’s test [32], which is a robust alternative to the traditional analysis of variance (ANOVA) [33].

## 4. Results

### 4.1. Cirque Size, Shape, and Development Degree

**Figure 3:**
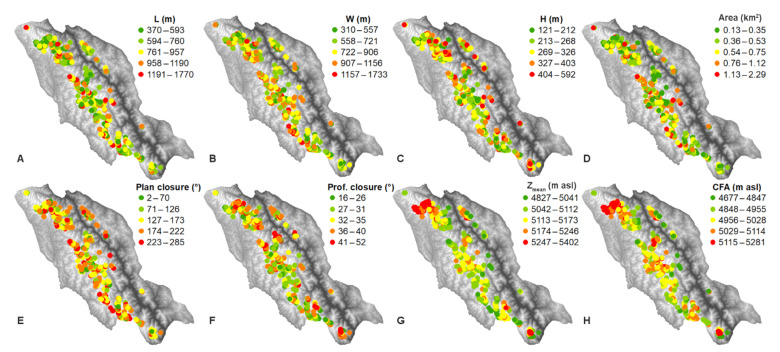
Spatial distributions of the cirque (**A**) L, (**B**) W, (**C**) H, (**D**) area, (**E**) plan closure, (**F**) profile closure, (**G**) Z_mean_, and (**H**) CFA.

A total of 361 cirques were mapped in the study area. The cirque L and W ranged from 370 to 1770 m and from 310 to 1733 m, with mean values of 787 and 795 m, respectively. The cirque H ranged from 121 to 592 m (mean: 293 m). The cirque area ranged from 0.13 to 2.29 km^2^, with a mean value of 0.56 km^2^ (Table 1). The spatial distributions of the cirque L, W, H, and area were consistent, which showed low values in the northernmost, southernmost, and northeastern parts of the study area (Figure 3A–D). The cirque L/W, L/H, and W/H ratios ranged from 0.46 to 2.15 (mean: 1.02), 1.25 to 4.72 (mean: 2.73), and 0.95 to 5.32 (mean: 2.80), respectively. The plan closure ranged from 2° to 285°, with a mean value of 162°, while the profile closure ranged from 16° to 52°, with a mean value of 33° (Table 1). The plan and profile closure values of the northern part were larger than those of the southern part (Figure 3E,F).

The cirque L, W, H, and area positively correlated with each other (0.53 ≤ *r* ≤ 0.88; *p* < 0.05). The cirque area was positively correlated with the L/H and W/H ratios (0.28 ≤ *r* ≤ 0.38; *p* < 0.05). The plan closure was positively correlated with the cirque L, W, area, L/H, and W/H (0.20 ≤ *r* ≤ 0.31; *p* < 0.05), while the profile closure was positively correlated with the cirque L, W, H, and area (0.30 ≤ *r* ≤ 0.53; *p* < 0.05), but it was negatively correlated with the L/H and W/H ratios (–0.19 ≤ *r* ≤ –0.13; *p* < 0.05). The cirque L, W, H, and area were positively correlated with the mean annual air temperature (MAAT) (0.17 ≤ *r* ≤ 0.29; *p* < 0.05) but negatively correlated with the mean annual precipitation (MAP) (–0.23 ≤ *r* ≤ –0.16; *p* < 0.05) (Table 2).

### 4.2. Cirque Altitude, Slope, and Aspect

The cirque Z_mean_ ranged from 4827 to 5402 m asl (mean: 5144 m asl). The CFA ranged from 4677 to 5281 m asl (mean: 5010 m asl). The cirque S_mean_ ranged from 14.55° to 35.46° (mean: 23.61°) (Table 1). The northern part of the study area had high Z_mean_ and CFA values. Some high values of Z_mean_ and CFA were present in the central and southern parts (Figure 3G,H). The spatial distributions of the Z_mean_ and CFA contrasted with those of the cirque size (L, W, H, and area) (Figure 3). The cirque Z_mean_ was negatively correlated with the cirque L, W, H, area, L/H, plan closure, and CFA (–0.38 ≤ *r* ≤ –0.14; *p* < 0.05), while the CFA was not correlated with the cirque size and shape metrics. The cirque S_mean_ was negatively correlated with the cirque L, W, area, L/H, W/H, and plan closure (–0.71 ≤ *r* ≤ –0.11; *p* < 0.05) and positively correlated with the cirque H and profile closure (0.37 ≤ *r* ≤ 0.45; *p* < 0.05). The cirque Z_mean_ was negatively correlated with the MAAT (*r* = –0.78; *p* < 0.05) but positively correlated with the MAP (*r* = 0.70; *p* < 0.05), while the CFA was positively correlated with the MAAT (*r* = 0.13; *p* < 0.05) (Table 2). With the increase in altitude, the cirque L, W, H, area, and plan closure decreased, while the L/W, L/H, profile closure, and CFA did not show a statistical difference between altitudinal groups at the 0.05 level (Table 3). With altitude, the S_mean_ first decreased from 25.69° to 23.12° and then increased to 24.70°. With altitude, the cirque aspect tended to shift along the clockwise direction from 357.90° to 126.75° (from N to SE), and the vector strength values first decreased from 72.95% to 30.80% and then increased to 66.77% (Table 3).

The dominant aspects for the cirques were NE and E (*n* = 82; 22.7%), followed by N (*n* = 63; 17.5%), while the SW was the least common aspect (*n* = 15; 4.2%). At the 0.05 level, the cirque profile closure, Z_mean_, and S_mean_ were statistically different between the aspects at the 0.05 level, while the L, W, H, area, L/W, L/H, W/H, plan closure, and CFA were not (Table 4). The cirques facing SE (mean: 34.73°) had the largest profile closure, followed by those facing E (mean: 34.29°) and NE (mean: 34.10°); those facing W had the smallest profile closure (mean: 29.73°). The cirques facing SE had the highest Z_mean_ (mean: 5214 m asl), followed by those facing S (mean: 5186 m asl) and SW (mean: 5178 m asl); those facing NW had the lowest (mean: 5114 m asl). The W-facing cirques had the largest S_mean_ (mean: 25.43°), followed by the NW-facing ones (mean: 24.66°); the SE-facing cirques had the smallest (mean: 22.64°) (Table 4).

### 4.3. Cirque Lithology

The cirques were distributed along a NW–SE axis, which was consistent with that of the Taniantaweng Range. A total of nine bedrock types were recognised, of which, phyllite/slate dominated (*n* = 152; 42.1%), followed by sand–slate (*n* = 63; 17.5%), monzonite (*n* = 38; 10.5%), and granodiorite (*n* = 33; 9.1%), while clastic with coal and intermediate-acid volcanic were rare (*n* = 12; 3.3%). The cirque L, W, H, area, L/H, W/H, profile closure, Z_mean_, CFA, and S_mean_ showed statistical differences between the bedrock groups at the 0.05 level, while the L/W and plan closure did not (Table 5). The cirques on the ‘other’ bedrocks had the largest L and W, while those on the marine clastic with coal had the smallest value. The largest cirque H was from those on the intermediate-acid volcanic, and the smallest value was from those on the clastic with coal. The cirques on ‘other’ bedrock had the largest area, while those on the clastic with coal had the smallest. The largest mean profile closure value was from the cirques on the marine clastic/limestone, and the smallest was from those on the syengranite. The cirques on the clastic with coal showed the highest Z_mean_, and those on the intermediate-acid volcanic showed the smallest. The highest and lowest CFAs were from those on the intermediate-acid volcanic and monzonite, respectively (Table 5).

## 5. Discussion

### 5.1. Impact of Climatic and Non-Climatic Factors on Cirques

The Last Glacial period (MIS 4–2) is assumed to have had a major effect on cirque development [2]. The climate of the TP during MIS 4 and 2 was characterised by strong westerlies and a weak ISM [34]. From west to east, the reconstructed temperature and precipitation during the Last Glacial Maximum (LGM, a period of MIS 2) of the study area decreased [21,35], which had a similar spatial pattern to the modern one (Figure 2). MIS 3 had a strong ISM and weak westerlies [34], which implies that the climatic pattern during MIS 3 was similar to the modern situation. Therefore, the spatial patterns of temperature and precipitation during the Last Glacial period were similar to the present, and the modern climatic pattern was used to analyse the relation of the cirque and the climate in the study area. The western part of the study area was characterised by mountain ranges and thus low temperatures. The study area was dominated by the ISM, which entered this region from the southwestern direction. Most cirques were located in the western part of the study area, where a cold and wet climate dominated (Figure 2 and Figure 3). This reveals the role of climate on the cirque locations. In contrast, few cirques were found in the eastern part of the study area due to the warm and arid climate. However, the cirque size (L, W, H, and area) increased with temperature and decreased with precipitation (Figure 2 and Figure 3A–D, Table 2), implying that large cirques tended to develop under a warm and dry climate. This was inconsistent with the findings of the Gangdise Mountains and the central TP, where the cirque sizes showed consistent spatial distributions with those of precipitation [6,7]. One explanation for this is that cirque enlargement is mainly limited to the duration when a cirque is occupied by a cirque-type glacier [2]; when low temperature and high precipitation levels surpass certain ranges, the glacier extends beyond the cirque boundary and limits cirque enlargement [7]. The positive correlation between CFA and MAAT implies that high temperature limits cirque development. However, this correlation is weak, and it implies that non-climatic factors affect CFA. The cirques tended to be located on leeward slopes because snow drifting from the windward slope promoted cirque development, and the enhanced heat exchange on the windward slope limited cirque development [28,36]. The cirques in this area tended to face NE and E (Table 5), and this may reveal this effect, because the ISM originated from the southwestern direction. The tendency of facing NE may also reveal the ‘morning–afternoon effect’, which documents that the polar tendency of the cirque aspects in the northern hemisphere often displace to the NE due to the coincidence of low solar radiation and low air temperatures in the morning [28,37].

The cirques tended to develop in the mountainous regions of the study area, where high altitudes dominated. This implies that local topography controlled the locations of the cirques. With altitude, the cirque aspects shifted from N to SE, implying that high altitude promoted cirque development on an unfavourable aspect, which is in line with the finding of the central TP [6]. The cirque size (L, W, H, and area) decreased with altitude, which is contrary to the results of the Gangdise Mountains and the central TP, where the cirque size increased with the altitude [6,7]. Two possible reasons were put forward for this result in this study area: (i) with altitude, temperature decreases and precipitation increases, which results in the development of valley-type glaciers and thus limits cirque enlargement; (ii) with altitude, the accommodation space for cirque enlargement is limited (cf. [25]). The negative correlation between the plan closure and Z_mean_ indicates that the cirques in high altitudes had a low development degree, which was in line with their small sizes. The CFA had a negative correlation with Z_mean_, which was contrary to the consensus that CFA increased with altitude. However, this correlation was weak (*r* = −0.10; *p* < 0.05). Furthermore, no statistical differences were found between altitudinal groups at the 0.05 level (Table 3), indicating that altitude did not play a major role in CFA.

The dominant cirque aspects of NE and E were partly caused by the NW–SE axis of the mountain orientation, which was in line with the results of the Gangdise Mountains [7] and the central TP [6]. The cirque size and shape did not show statistical differences between the aspects (Table 4), indicating that the aspect did not affect the cirque size and shape. This implies that the ISM had a limited effect on the cirque size and shape. If NW, N, and NE are taken as the ‘northern slope’ and SW, S, and SE as ‘southern slope’, the profile closures of the cirques on the ‘northern slope’ were larger than those on the ‘southern slope’, indicating that cirques on the poleward slope were better developed in the vertical direction. If NE, E, and SE are taken as the ‘eastern slope’ and NW, W, and SW as the ‘western slope’, then the larger profile closure for the cirques on the eastern slope may reflect that the cirques on the leeward slope were better developed in the vertical direction. The cirques located on the ‘southern slope’ had a higher altitude than those on the ‘northern slope’, implying that high solar radiation levels led to cirque development at a high altitude.

Cirques on soft bedrocks usually tend to be larger and have lower CFAs than those on hard bedrocks [6,7,16]. Indeed, the cirques on sand–slate, marine clastic/limestone, and clastic with coal (i.e., soft bedrocks) had a larger H than those on monzonite, granodiorite, and syengranite (i.e., hard bedrocks) (Table 5), which is in line with the findings mentioned above. However, the cirques on the soft bedrocks had a smaller L, W, and area than those on the hard bedrocks (Table 5). This implies that soft bedrocks promote subglacial erosion other than rock wall erosion.

### 5.2. Monsoon’s Role on Cirques

The MAAT and MAP values of the central and southeastern TP and the Gangdise Mountains showed that the strength of the ISM was greatest in the southeastern TP and then decreased westward to the central–eastern Gangdise Mountains, whilst the western Gangdise Mountains and the central TP were dominated by a continental climate with coldness and aridity (Table 6) [6,7,16]. The cirque density of the study area was 0.043 n km^–2^, which was larger than those of the central TP and the western sector of the Gangdise Mountains but smaller than those of the central and eastern sectors of the Gangdise Mountains (Table 6). This does not support the suggestion that with the strengthening of the ISM (i.e., temperature and precipitation increase), cirque density increases [6]. One reason is that the southeastern TP was the most strongly ISM-dominated region on the TP; this result may imply that when the monsoon strength (temperature and precipitation levels) surpasses a certain range, cirque development is limited. Another reason is that cirque density is affected by non-climatic factors (e.g., topography and accommodation space), and there is no clear relation between monsoon strength and cirque density.

The mean values of the cirque L and H of this study were smaller than those of the central TP and the western, central, and eastern sectors of the Gangdise Mountains. The mean value of the cirque W of this study was smaller than those of the central TP, western, and central sectors of the Gangdise Mountains, but it was larger than that of the eastern sector of the Gangdise Mountains. The mean cirque area was smaller than those of the central TP, the western, and central sectors of the Gangdise Mountains, but it was slightly larger than that of the eastern sector of the Gangdise Mountains (Table 6) [6,7]. It was found that the cirques in the ISM-dominated regions had smaller sizes than those in the continental climate, indicating that a strengthened ISM limits cirque size in a regional scale. This finding is in line with that of Zhang et al. [6]. The CFA was highest in the central sector of the Gangdise Mountains, followed by the western and eastern sectors of the Gangdise and the central TP, while the mean CFA in the southeastern TP was the lowest. Among the five regions, the western sector of the Gangdise Mountains and the central TP receives the least monsoonal moisture, but they do not have the highest CFAs. The spatial trend of the CFA was consistent with that of the Z_mean_ in these regions, implying that the spatial trend of the CFA is greatly affected by cirque altitude at a regional scale. This indicates that the CFA does not necessarily reflect climate, which supports the finding of the Gangdise Mountains [16].

## 6. Conclusions

A total of 361 cirques were mapped in the Taniantaweng Range of the southeastern TP, and their metrics were calculated. The cirques tended to be located in the mountainous areas in the western part of the study area. The cirque size (L, W, H, and area) increased with temperature and decreased with precipitation, implying that large cirques tended to develop in a warm and dry climate. The cirques tended to face NE, implying that they prefer leeward slopes, and they were under the ‘morning–afternoon’ effect. With altitude, the tendency of the cirque aspect shifted from N to SE, and the cirque size decreased. The former may indicate the ability of a high altitude to support cirque development on climatically unfavourable slopes; the latter may be due to the development of valley-type glaciers or insufficient space for cirque development. The negative correlation between plan closure and Z_mean_ implies that the cirque at a high altitude had a small development degree, while the weak correlation between CFA and Z_mean_ implies that altitude does not play a major role in the CFA on a local scale. The cirque size and shape did not show statistical differences between aspects, which implies that on a local scale the effect of the ISM on cirque size and shape is limited. The cirques on soft bedrocks had a larger H than those on hard bedrocks, indicating that soft bedrocks promote subglacial erosion.

The cirque density of the southeastern TP was larger than those of the western sector of the Gangdise Mountains and the central TP but smaller than those of the central–eastern sectors of the Gangdise Mountains. This indicates that with the strength of the ISM, cirque density does not necessarily increase, which is likely due to the fact that a strengthened ISM limits cirque development (i.e., glaciers extended beyond cirque boundaries and developed into valley-type glaciers, thus limiting cirque development), or cirque density is affected by non-climatic factors. The cirques in the central–eastern sectors of the Gangdise Mountains and the southeastern TP had smaller sizes than those in the western sector of the Gangdise Mountains and the central TP, which implies that on a regional scale, a strengthened ISM limits cirque size. The comparison of the CFA in the western, central, and eastern sectors of the Gangdise Mountains, central TP, and the southeastern TP reveals that the CFA did not show a reverse relationship with precipitation, but shows a positive correlation with the cirque Z_mean_, which implies that the CFA is greatly affected by altitude on a regional scale, and its distribution does not always reflect paleoclimatic patterns.

## Figures and Tables

**Table 1:** Descriptive statistics of the cirque metrics of the study area ^a^.

	Minimum	Maximum	Mean	Std. Deviation	Skewness
L (m)	370	1770	787	248.21	0.96
W (m)	310	1733	795	234.84	0.68
H (m)	121	592	293	77.43	0.58
Area (km^2^)	0.13	2.29	0.56	0.31	1.47
L/W	0.46	2.15	1.02	0.28	0.60
L/H	1.25	4.72	2.73	0.64	0.63
W/H	0.95	5.32	2.80	0.77	0.66
Plan closure (°)	2	285	162	61.33	−0.51
Profile closure (°)	16	52	33	6.12	0.29
Z_mean_ (m asl)	4827	5402	5144	86.01	0.25
CFA (m asl)	4677	5281	5010	102.65	−0.10
S_mean_ (°)	14.55	35.46	23.61	3.44	0.52

^a^ L: length; W: width; H: height; L/W: ratio of length to width; L/H: ratio of length to height; W/H: ratio of width to height; Z_mean_: mean altitude; CFA: cirque floor altitude; S_mean_: mean slope gradient.

**Table 2:** Pearson’s r of the cirque metrics of the study area ^a^. The correlations in bold are significant at the 0.05 level.

Log of	L	W	H	Area	L/W	L/H	W/H	Plan Closure	Profile Closure	Z_mean_	CFA	S_mean_	MAAT	MAP
L	1.00	**0.58**	**0.67**	**0.88**	**0.47**	**0.53**	−0.03	**0.28**	**0.37**	**−0.35**	0.07	**−0.15**	**0.26**	**−0.23**
W		1.00	**0.53**	**0.87**	**−0.45**	**0.15**	**0.57**	**0.27**	**0.30**	**−0.33**	−0.01	**−0.12**	**0.17**	**−0.16**
H			1.00	**0.67**	**0.17**	**−0.27**	**−0.40**	0.09	**0.53**	**−0.28**	0.04	**0.45**	**0.29**	**−0.22**
Area				1.00	0.03	**0.38**	**0.28**	**0.31**	**0.38**	**−0.38**	0.04	**−0.17**	**0.23**	**−0.21**
L/W					1.00	**0.42**	**−0.65**	0.02	0.08	−0.03	0.08	−0.03	**0.10**	−0.08
L/H						1.00	**0.42**	**0.26**	**−0.13**	**−0.14**	0.04	**−0.71**	0.02	−0.05
W/H							1.00	**0.20**	**−0.19**	−0.09	−0.05	**−0.56**	−0.09	0.04
Plan closure								1.00	0.08	**−0.23**	0.04	**−0.11**	**0.14**	**−0.14**
Profile closure									1.00	0.07	−0.01	**0.37**	0.06	0.02
Z_mean_										1.00	**−0.10**	0.10	**−0.78**	**0.70**
CFA											1.00	0.07	**0.13**	−0.04
S_mean_												1.00	0.04	0.01
MAAT													1.00	**−0.73**
MAP														1.00

^a^ See Table 1 for attribute definition.

**Table 3:** Cirque metrics against altitude ^a^.

Altitude (m asl)	Nr	L (m)	W (m)	H (m)	Area (km^2^)	L/W	L/H	W/H	Plan Closure (°)	Profile Closure (°)	Z_mean_ (m asl)	CFA (m asl)	S_mean_ (°)	VM (°)	VS (%)
≤5000	14	952	868	377	0.72	1.10	2.56	2.41	193.00	32.68	4960	5086	25.69	357.90	72.95
5001–5100	97	887	867	318	0.69	1.05	2.82	2.82	174.97	33.10	5066	5012	23.34	15.47	43.33
5101–5200	166	770	812	281	0.55	0.98	2.77	2.95	160.68	32.50	5144	5008	23.12	43.08	45.28
5201–5300	69	689	683	275	0.42	1.06	2.59	2.56	148.52	34.46	5246	4997	24.54	60.01	30.80
≥5301	15	626	600	263	0.32	1.10	2.51	2.43	114.53	34.73	5343	5004	24.70	126.75	66.77
*p*-value ^b^		0.000	0.000	0.000	0.000	0.107	0.054	0.000	0.001	0.236	0.000	0.133	0.003		

^a^ See Table 1 for attribute definition. ^b^ By Welch’s test.

**Table 4:** Cirque metrics against aspect ^a^.

Aspect	Nr	L (m)	W (m)	H (m)	Area (km^2^)	L/W	L/H	W/H	Plan Closure (°)	Profile Closure (°)	Z_mean_ (m asl)	CFA (m asl)	S_mean_ (°)
N	63	736	762	278	0.50	1.00	2.70	2.81	175.54	32.72	5121	5018	23.85
NE	82	785	811	297	0.56	1.02	2.68	2.80	155.40	34.10	5128	5007	23.36
E	82	859	808	299	0.63	1.07	2.90	2.79	154.87	34.29	5159	4987	22.88
SE	26	802	788	291	0.57	1.05	2.85	2.80	133.12	34.73	5214	5025	22.64
S	18	747	756	267	0.50	1.03	2.80	2.87	170.50	31.84	5186	5020	23.32
SW	15	806	860	311	0.61	1.03	2.67	2.92	164.67	31.89	5178	5005	23.29
W	30	790	818	309	0.58	0.98	2.61	2.73	174.07	29.73	5134	5027	25.43
NW	45	730	774	290	0.52	0.98	2.57	2.75	168.67	32.14	5114	5025	24.66
*p*-value ^b^		0.111	0.826	0.282	0.381	0.517	0.127	0.998	0.073	0.007	0.000	0.420	0.010

^a^ See Table 1 for attribute definition. ^b^ By Welch’s test.

**Table 5:** Cirque metrics against bedrock types ^a^.

Bedrock Types	Nr	L (m)	W (m)	H (m)	Area (km^2^)	L/W	L/H	W/H	Plan Closure (°)	Profile Closure (°)	Z_mean_ (m asl)	CFA (m asl)	S_mean_ (°)
Phyllite/slate	152	785	811	280	0.57	1.00	2.84	2.95	159.01	32.58	5146	5003	22.99
Sand–slate	63	786	780	325	0.54	1.04	2.45	2.46	168.17	34.30	5140	5029	25.28
Monzonite	38	778	754	289	0.52	1.06	2.73	2.72	162.55	31.90	5106	4963	22.53
Granodiorite	33	761	765	294	0.52	1.04	2.69	2.71	154.55	33.82	5118	4965	24.33
Other	23	944	1002	296	0.86	0.93	3.12	3.40	193.17	31.67	5100	5073	22.07
Marine clastic/limestone	14	747	659	332	0.43	1.19	2.29	2.07	147.21	41.36	5234	5067	27.11
Syengranite	14	750	744	261	0.51	1.02	2.85	2.97	157.07	30.18	5235	4984	22.74
Clastic with coal	12	617	722	237	0.39	0.91	2.68	3.04	134.92	32.80	5289	5021	23.06
Intermediate-acid volcanic	12	881	790	337	0.60	1.13	2.68	2.40	162.50	33.16	5052	5110	24.69
*p*-value ^b^		0.018	0.004	0.001	0.004	0.085	0.000	0.000	0.274	0.000	0.000	0.000	0.000

^a^ See Table 1 for attribute definition. ^b^ By Welch’s test.

**Table 6:** Comparison of the cirque metrics (mean values) of the Gangdise Mountains, central TP, and this study ^a^.

	Central TP	W Gangdise Mountains	C Gangdise Mountains	E Gangdise Mountains	Southeastern TP
MAAT (°C)	−10 to 1	~−14 to 4	~−11 to 3	~−8 to 5	−5.2 to 9.2
MAP (mm)	110 to 230	~80 to 460	~130 to 310	~230 to 420	530 to 740
Density (n km^–2^)	0.006	0.036	0.072	0.187	0.043
L (m)	984	1033	960	815	787
W (m)	860	1014	838	741	795
H (m)	370	367	365	330	293
Area (km^2^)	0.78	0.94	0.74	0.55	0.56
L/W	1.21	1.06	1.18	1.13	1.02
L/H	2.78	2.85	2.65	2.49	2.73
W/H	2.43	2.86	2.39	2.32	2.80
Plan closure (°)	157	/	/	/	162
Profile closure (°)	59	/	/	/	33
Z_mean_ (m asl)	/	5676	5776	5526	5144
CFA (m asl)	5352	5508	5613	5371	5010
S_mean_ (°)	/	22	24	25	23.61
Sources	[6]	[7]			This study

^a^ See Table 1 for attribute definition.

## Supplementary Materials

The following supporting information can be downloaded at: https://www.mdpi.com/article/10.3390/ijerph192013104/s1, Cirque metrics.
